# Shifting Preference between Oviposition vs. Host-Feeding under Changing Host Densities in Two Aphelinid Parasitoids

**DOI:** 10.1371/journal.pone.0041189

**Published:** 2012-07-17

**Authors:** Nian-Wan Yang, Lu-Lu Ji, Gabor L. Lövei, Fang-Hao Wan

**Affiliations:** 1 State Key Laboratory for Biology of Plant Diseases and Insect Pests, Institute of Plant Protection, Chinese Academy of Agricultural Sciences, Beijing, People's Republic of China; 2 Department of Agroecology, Aarhus University, Flakkebjerg, Denmark; California State University Fullerton, United States of America

## Abstract

Destructive host-feeding is common in hymenopteran parasitoids. Such feeding may be restricted to host stages not preferred for oviposition. However, whether this is a fixed strategy or can vary according to resource levels or parasitoid needs is less clear. We tested the trade-off between host feeding and oviposition on two whitefly parasitoids under varying host densities. Females of two aphelinid parasitoids, *Eretmocerus hayati* and *Encarsia sophia* were exposed to nine different densities of their whitefly host, *Bemisia tabaci*, in single-instar tests to identify their functional response. Mixed-instar host choice tests were also conducted by exposing whiteflies at four densities to the parasitoids. We hypothesized that the parasitoid females can detect different host densities, and decide on oviposition vs. host-feeding accordingly. The results showed that both *Er. hayati* and *En. sophia* females tended to increase both oviposition and host-feeding with increased host density within a certain range. Oviposition reached a plateau at lower host density than host-feeding in *Er. hayati*, while *En. sophia* reached its oviposition plateau at higher densities. At low densities, *Er. hayati* parasitized most on first and second (the optimal ones), and fed most on third nymphal instars (the suboptimal one) of the whitefly host as theory predicts, while at high densities, both parasitism and host-feeding occurred on first and second instars which are preferred for oviposition. *En. sophia* parasitized most on third and fourth (the optimal ones), while fed on first instars (the suboptimal one) at low densities, and utilized third and fourth instars for both at high densities. In conclusion, oviposition vs. host-feeding strategy of parasitoid females was found to vary at different host densities. The balance between reserving optimal hosts for oviposition or using them for host-feeding depended on parasitoid life history and the availability of host resources.

## Introduction

Parasitoids have attracted considerable attention because of their importance in biological control of pests, but also due to their value as experimental models in investigating the evolution of reproductive strategies. When confronting a host, the parasitoid females make a series of decisions that have major consequences for the fitness of their offspring [1]. Besides oviposition, many parasitoid species use hosts for food as well. This host-feeding behaviour has been observed in more than 140 species belonging to 17 hymenopteran families [2]. Host feeding provides nutrients for maturing eggs [2]–[4] and prolongs female longevity [3], [5] potentially increasing future reproduction.

On the other hand, host-feeding kills the host or at least reduces the quality of the host for oviposition, and the host handling time is often longer than that of ovipositing, increasing the risk of predation to the parasitoid [4]. On encountering a host, the parasitoid should decide whether to oviposit or to host-feed. This decision is affected by factors related to the physiological state of the parasitoid, such as egg load, nutritional status and life expectancy, as well as factors related to host quality and availability [6]–[9].

Optimal foraging models predict that host-feeding parasitoid females, when attacking different types of hosts of varying quality, should oviposit on hosts of high quality, while feeding on ones of lower quality [3], [10], resulting in more efficient use of host resources [11]. Such behaviour is reported in several species, including *Encarsia formosa* (Gahan) (Hymenoptera: Aphelinidae) [12], *Aphytis lingnanensis* (Compere) (Hymenoptera: Aphelinidae) [6], and *Diadromus subtilicornis* (Gravenhorst) (Hymenoptera: Ichneumonidae) [13]. However, these studies were conducted either in no-choice experiments involving a single host stage, or in mixed-host-stage choice-experiment at constant host densities. The question whether host stage preference for ovposition vs. host-feeding varies with host density has not yet been explored.

In order to better understand host instar allocation for oviposition vs. host-feeding in response to different host densities, we exposed two aphelinid parasitoids to a variable density host system. Single-instar no choice tests with different host densities were conducted to establish the functional response of both behaviours. Subsequently, mixed-instar host choice tests were conducted to test the host instar allocation ability of the parasitoid females. We hypothesized that the parasitoid females can detect different host densities, and vary their oviposition vs. host-feeding accordingly. Under abundance of high quality hosts (at high host density), a parasitoid female is expected to both oviposit and host-feed on its favourite host stages. When host resource is limited (at low host density and/or quality), a parasitoid female would refrain from host feeding on the optimal host instar for oviposition, and feed on alternative host instars.

## Materials and Methods

### Study system

Two parasitoid species, *Encarsia sophia* (Girault & Dodd) (Hymenoptera: Aphelinidae) and *Eretmocerus hayati* (Zolnerowich & Rose) (Hymenoptera: Aphelinidae), are good candidates to be used as biological control agents for *Bemisia tabaci* (Gennadius) (Homoptera: Aleyrodidae) Middle East-Asia Minor 1 (formerly also known as “biotype B”) which is one of the most important invasive insect pests in China [14]–[17]. *En. sophia* (formerly also known as *Encarsia transvena* (Timberlake)) is a solitary, heteronomous hyperparasitoid. Female-producing (fertilized) eggs are laid internally in whitefly nymphs and develop as primary parasitoids, whereas male-producing (unfertilized) eggs are laid in the body fluids of a previously parasitized whitefly nymphs, by their own or of other *Encarsia* and *Eretmocerus* spp. [18], [19], and develop as hyperparasitoids.

*Er. hayati* is a solitary parasitoid ovipositing externally under whitefly nymphs [20]. Upon eclosion, the first instar larva penetrates the host cuticle, feeds and pupates internally. This species is a biparental primary parasitoid, with both males and females developing in whitefly nymphs.

Both *En. sophia* and *Er. hayati* females oviposit and host-feed on all nymphal instars of *B. tabaci* with the exception of late fourth instars. Third and fourth instars are the optimal hosts for *En. sophia* oviposition, while *Er. hayati* oviposits mostly on first and second instars [15], [21].

### Stock cultures of insects and host plants

Laboratory colonies of *Er. hayati* and *En. sophia* in China were established from parasitized *B. tabaci* populations maintained on melon plants in a greenhouse in the Vegetable IPM Laboratory, Texas Agricultural Experiment Station at Weslaco, TX, USA. A stock culture of *B. tabaci* Middle East-Asia Minor 1 [17] was established using 300 individuals from a colony which had been maintained on tomato plants for the last 2 years without any exposure to pesticides, and obtained from the Institute of Vegetables and Flowers, Chinese Academy of Agricultural Sciences (CAAS).

Whiteflies and parasitoids were maintained on tomato plants, *Solanum lycopersicum* L. var. *lycopersicum* (Solanaceae), variety Zhong-Za No. 9, in an air conditioned glasshouse, at 26±2°C, and a natural light regime (39°57′ N, 116°19′ E), at the Institute of Plant Protection, CAAS, Beijing, China. Plants reached approximately 15 cm height with 5–7 fully expanded leaves were used.

### Experimental parasitoids

*B. tabaci*-infested tomato plants were exposed to naïve female wasps (up to 1-day old) for 24 h. Subsequently, plants were maintained in an air-conditioned laboratory, where all experiments were conducted, at 26±1°C, 65±5% RH and a light regime of 14:10 (hours L:D). After 13 days, parasitoid pupae were collected and individually put in a Petri dish with a drop of honey (5%). Petri dishes were checked daily for emergence. Females that emerged were provided with males and were observed mating, then used in pair in experiments 1 day later. All experimental females had no ovipositing experience.

### Single-instar no-choice tests

In these experiments, only the single (optimal) nymphal instar of *B. tabaci* was offered to the parasitoids: second instar nymphs (N2) to *Er. hayati*, and third instar nymphs (N3) to *En. sophia* females. In order to generate the required nymphal population, 10, 20, 30 or 40 unsexed whitefly adults were introduced into a clip cage (transparent plastic cup, base diameter 2.1 cm) on a tomato leaf (one clip cage per plant) for a 12-h oviposition period to assure host stage uniformity. The adults were removed and the eggs were monitored daily until they developed to the desired stage (N2 or N3). Five, 10, 20, 30, 40, 50, 60, 70 or 80 nymphs were left on each leaf in the area covered by a clip cage (3.5 cm^2^). A pair of 1 d-old *Er. hayati* or *En. sophia* was introduced into a clip cage confining the nymphs in each of the nine host densities for a 24-h oviposition and feeding period, after which they were removed. The numbers of whitefly hosts that were killed by parasitizing or host-feeding were checked 7–8 days after parasitoid removal. If the hosts were parasitized, the mycetome displacement was visible through the cuticle at the time of examination [22]. If the hosts were killed by host-feeding, the bodies became flat, desiccated and colour faded [23]. The hosts killed by attempting (but failed) host-feeding or dead naturally, identified by the appearance of bodies that contain inclusions and turn darker because of no/less body fluid is lost, were excluded from the count. The experiment had a total of 18 treatments (nine host densities and two parasitoid species). A total of 15 pairs for each treatment were initially used. The replicates in which the introduced parasitoids escaped or died were excluded, and the data for at least10 replicates were used in data analysis.

### Mixed-instar host choice tests

Ten to 20 unsexed whitefly adults were introduced into a clip cage on a tomato leaf (one clip cage per plant) for a 12-h oviposition period, then removed to assure stage uniformity. The whitefly adults were introduced on the same tomato leaf for 1, 3 and 6 days after the first removal of the introduced adults, respectively. The whitefly eggs were monitored daily until they developed to the desired stage, namely first (N1), second (N2), third (N3) and early fourth instars (N4). The four nymphal instars of *B. tabaci* were offered to the parasitoids simultaneously. A total of 20, 40, 60 and 80 nymphs with different instars in equal proportions were left on each leaf in the area and covered by a clip cage (3.5 cm^2^). The distributions of the nymphs on each experimental plant leaf were photographed under a stereomicroscope and the photos were printed out. A pair of 1 d-old *Er. hayati* or *En. sophia* adults was introduced into a clip cage confining the nymphs in each of the four host densities for a 24-h oviposition and feeding period, then removed. The numbers of whitefly hosts that were killed by parasitizing and host-feeding were checked 7–8 days after parasitoid removal. The host instars killed by parasitizing or feeding were determined by using the photos taken before parasitoid introduction. The experiment had a total of 8 treatments (four host densities and two parasitoid species). A total of 15 pairs for each treatment were initially used. The replicates in which the introduced parasitoids escaped or died were abandoned. The data for at least10 replicates were used in data analysis.

### Statistical analysis

In single-instar no choice experiment, the differences in the number of whitefly hosts parasitized, as well as the number of whitefly hosts fed by both species of parasitoids were analyzed using one-way ANOVA (SPSS version 13.0 software package) with the factor of host density. Differences among means related to different host densities were compared with Tukey's Honestly Significant Difference (HSD) test at the significance level p = 0.05.

In mixed-instar choice experiment, a two-way ANOVA was used, with whitefly host instar and host density as factors, to analyze their effects on mean number of hosts killed (the sum of hosts parasitized or host-fed), parasitized, as well as host-fed. If the factor showed significant influence, the differences in the number of whitefly hosts killed, parasitized, or host-fed at each host density were analyzed using one-way ANOVA with the factor of host instar. Differences in the number of whitefly hosts killed, parasitized, or host-fed on each host instar were also analyzed by one-way ANOVA with the factor of host density. Differences among means related to different host instars or different host densities were compared with HSD test at p = 0.05.

The host-feeding ratio was defined as:

Host-feeding ratio  =  no. of hosts fed upon/(no. of host parasitized + no. of host fed upon).

In both single-instar and mixed-instar experiments, differences among means of host-feeding ratio of both species of parasitoids related to the factor of different host densities were compared with Kruskal-Wallis test at p = 0.05. In mixed-instar experiment, differences among means of host-feeding ratio of both species of parasitoids on different host instars were also compared with Kruskal-Wallis test at p = 0.05 with the factor of host instar.

Differences between means of host-feeding ratio by the factor of different species of parasitoids at each host density in single-instar experiment, as well as the differences between means of host-feeding ratio in single-instar experiment and means of host feeding ratio on each instars in total in mixed-instar experiment with the factor of no-choice or choice condition were compared with Mann-Whitney test at p = 0.05. We used non-parametric methods because host-feeding ratio did not meet the assumption of normal distribution and equal variances for parametric method even after transformation.

## Results

### Single-instar no choice tests

**Figure 1:**
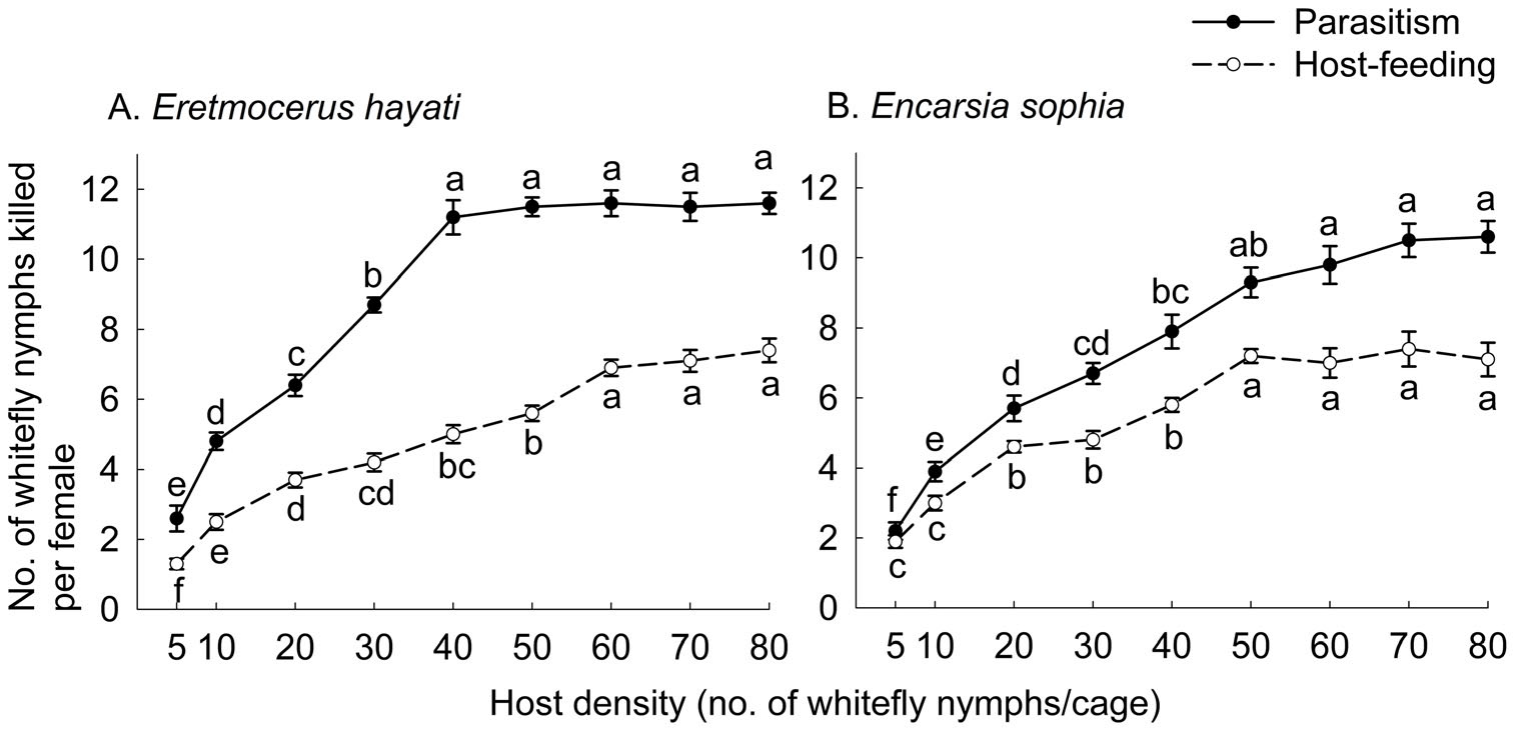
Mean number of whitefly nymphs killed by parasitism or host-feeding by parasitoid in single-instar experiment. A, B: killed by 1 d-old *Eretmocerus hayati* or *Encarsia sophia* in 24 h, respectively. An area of 3.5 cm^2^ of a leaf on a potted tomato plant was covered by a clip cage. Data points with different lowercase letters in each line indicate significant differences (HSD test; *P*<0.05) in number of hosts parasitized (continuous line) or fed (broken line). Vertical bars indicate ± one SE.

The number of hosts parasitized by *Er. hayati* significantly increased with increased density of *B. tabaci* nymphs (*F*_8, 81_  = 103.70, *P*<0.0001) and reached a plateau at 11.2 (SE  = 0.5) at 40 2^nd^ instar nymphs per cage (Figure 1A). The number of hosts fed by *Er. hayati* also significantly increased with increased host density (*F*_8, 81_  = 72.05, *P*<0.0001) and reached a plateau at 6.9 (SE  = 0.2) at 60 nymphs per cage (Figure 1A).

The number of hosts parasitized by *En. sophia* significantly increased with increased *B. tabaci* nymphal density (*F*_8, 81_  = 54.43, *P*<0.0001) and reached the plateau level at 9.8 (SE  = 0.4) at 60 3^rd^ instar nymphs per cage (Figure 1B). The number of hosts fed by *Er. hayati* also significantly increased with increased host density (*F*_8, 81_  = 39.62, *P*<0.0001) and reached a plateau at 7.2 (SE  = 0.2) at 50 nymphs per cage (Figure 1B).

### Mixed-instar host choice tests

In mixed- instar host test, number of hosts parasitized, number of hosts fed, and the total number of hosts killed by parasitism and host-feeding were significantly affected by host instars and host densities (Table 1). There were significant interactions between host instars and host densities on parasitism and host-feeding of the two parasitoids (Table 1).

**Table 1:** The results of ANOVA of whitefly host instar and host density for parasitoids.

Source	*df*	Mean Square	*F*	*P*
***Eretmocerus hayati***				
No. of whitefly nymphs killed by parasitism and host-feeding				
Host instar	3	170.956	129.772	<0.0001
Host density	3	31.906	24.220	<0.0001
Host instar × host density	9	13.073	9.924	<0.0001
Error	144	1.317		
Total	160			
No. of whitefly nymphs killed by parasitism				
Host instar	3	78.850	113.092	<0.0001
Host density	3	10.383	14.892	<0.0001
Host instar × host density	9	2.256	3.235	0.001
Error	144	0.697		
Total	160			
No. of whitefly nymphs killed by host-feeding				
Host instar	3	19.423	27.023	<0.0001
Host density	3	5.906	8.217	<0.0001
Host instar × host density	9	4.828	6.718	<0.0001
Error	144	0.719		
Total	160			
***Encarsia sophia***				
No. of whitefly nymphs killed by parasitism and host-feeding				
Host instar	3	103.708	90.950	<0.0001
Host density	3	29.292	25.688	<0.0001
Host instar × host density	9	19.953	17.498	<0.0001
Error	144	1.140		
Total	160			
No. of whitefly nymphs killed by parasitism				
Host instar	3	61.108	89.975	<0.0001
Host density	3	11.842	17.436	<0.0001
Host instar × host density	9	4.347	6.401	<0.0001
Error	144	0.679		
Total	160			
No. of whitefly nymphs killed by host-feeding				
Host instar	3	7.217	10.561	<0.0001
Host density	3	4.150	6.073	0.001
Host instar × host density	9	6.567	9.610	<0.0001
Error	144	0.683		
Total	160			

#### Hosts parasitized and fed by Eretmocerus hayati

**Figure 2:**
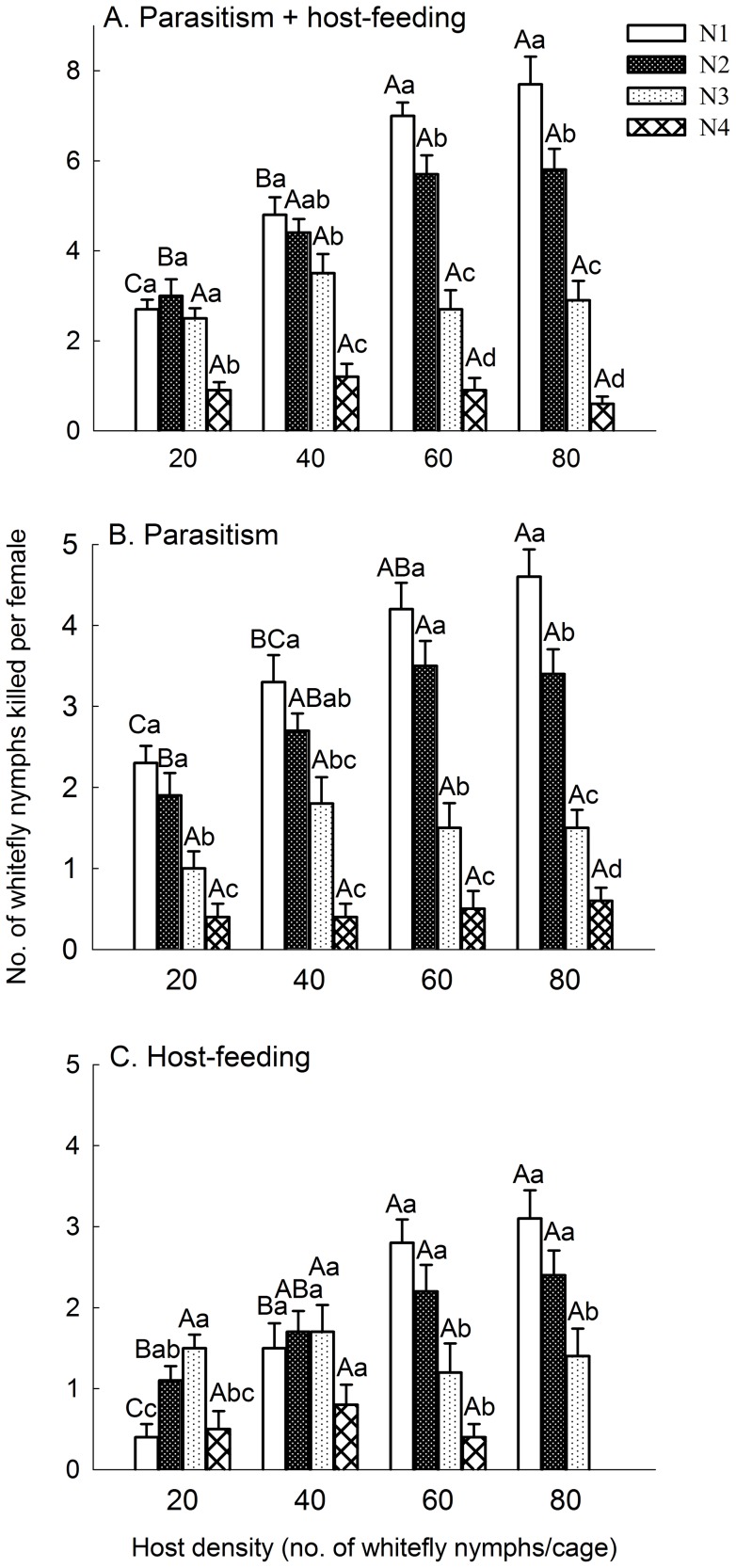
Mean number of whitefly nymphs killed by *Eretmocerus hayati* in mixed-instar hosts experiment. A: killed by parasitism and host-feeding in 24 h; B: killed by parasitism in 24 h; C: killed by host-feeding in 24 h. An area of 3.5 cm^2^ of a leaf on a potted tomato plant was covered by a clip cage. Host instars, N1, N2, N3 and N4 are first, second, third and early fourth instars, respectively. Bar heads with different lowercase letters in each cluster indicate significant differences (HSD test; *P*<0.05) in number of hosts parasitized or fed among different host instars. Bar heads with different capital letters for each instars between the clusters indicate significant differences (HSD test; P<0.05) in number of hosts killed among different host densities. Vertical bars indicate ± one SE.

In mixed-instar host test, the total number of whitefly hosts killed by parasitism and host-feeding by *Er. hayati* females varied significantly with host instars at all of the four host densities (Figure 2A, *F*_3, 36_  = 13.52, 20.27, 59.03 and 48.41 at 20, 40, 60 and 80 nymphs per cage, respectively, *P*<0.0001 for each). At 20 nymphs per cage, females killed a similar number of first, second and third host instar nymphs, and significantly fewer fourth instar ones. With the host density increasing to 40, 60 and 80 nymphs per cage, the first (*F*_3, 36_  = 30.92, *P*<0.0001) and second (*F*_3, 36_  = 11.10, *P*<0.0001) instar nymphs killed by females increased significantly, while the number of third (*F*_3, 36_  = 1.24, *P* = 0.308) and fourth (*F*_3, 36_  = 1.09, *P*  = 0.365) instar nymphs changed less (Figure 2A). At 60 and 80 nymphs per cage, female killed most on first instars, followed by second, third and fourth instars, in this sequence (Figure 2A).

The numbers of hosts parasitized by *Er. hayati* females varied significantly with host instar at all of the four host densities (Figure 2B, *F*_3, 36_  = 15.31, 21.85, 34.22 and 45.89, at 20, 40, 60 and 80 nymphs per cage, respectively, *P*<0.0001 for each). At all experimental host densities, *Er. hayati* parasitized significantly more first and second instars than third and fourth instars, with the exception that the number of third instars parasitized was not significantly less than that of second instars at 40 nymphs per cage (Figure 2B). The numbers of parasitized first (*F*_3, 36_  = 11.02, *P*<0.0001) and second instar nymphs (*F*_3, 36_  = 7.09, *P*  = 0.0007) increased significantly with increased host density, while the numbers of parasitized third (*F*_3, 36_  = 1.49, *P* = 0.234) and fourth instar nymphs (*F*_3, 36_  = 0.28, *P* = 0.838) did not (Figure 2B).

At the host density of 20 nymphs per cage, the number of different host instars fed by *Er. hayati* females also varied significantly (*F*_3, 36_  = 7.89, *P* = 0.0004): females fed significantly more number of third instars than first and fourth instars, and more second instars than first instars; there was no difference between the number of second and third instars fed by females (Figure 2C). At 40 nymphs per cage, there were no significant differences among the number of the four different instars fed (Figure 2C, *F*_3, 36_  = 2.17, *P* = 0.109), although the number of second and third instars fed was higher than the others. With the host density increased, the number of first (*F*_3, 36_  = 18.98, *P*<0.0001) and second instar nymphs (*F*_3, 36_  = 4.49, *P* = 0.009) fed by females increased significantly, while the number of third (*F*_3, 36_  = 0.45, *P* = 0.718) and fourth instar nymphs (*F*_3, 36_  = 3.144, *P* = 0.037) did not (Figure 2C). At 80 nymphs per cage, no fourth instar nymphs were fed upon (Figure 2C). At 60 (*F*_3, 36_  = 13.04, *P*<0.0001) and 80 (*F*_2, 27_  = 6.636, *P* = 0.005) nymphs per cage, *Er. hayati* female fed most on first and second instar nymphs (Figure 2C).

#### Hosts parasitized and fed by Encarsia Sophia

**Figure 3:**
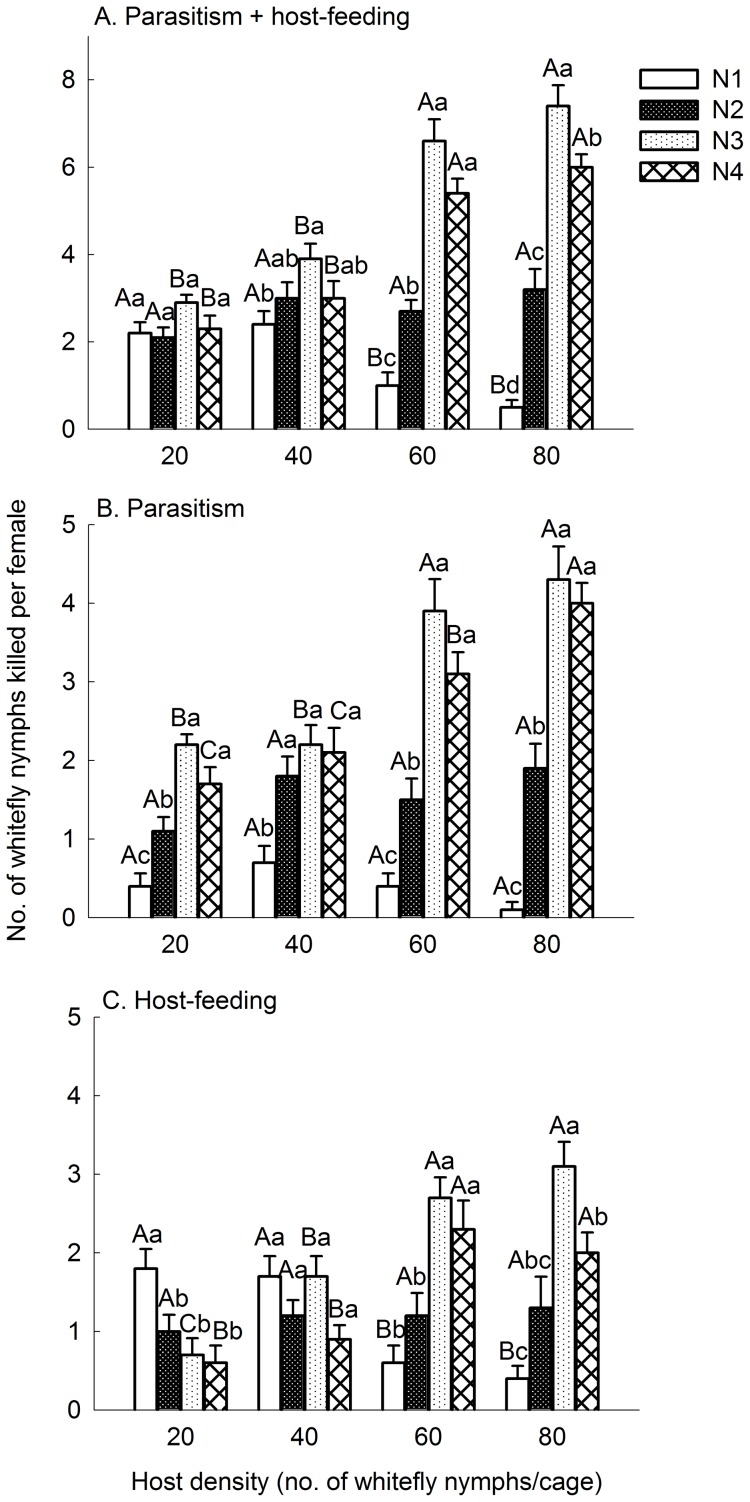
Mean number of whitefly nymphs killed *Encarsia sophia* in mixed-instar hosts experiment. A: killed by parasitism and host-feeding in 24 h; B: killed by parasitism in 24 h; C: killed by host-feeding in 24 h. An area of 3.5 cm^2^ of a leaf on a potted tomato plant was covered by a clip cage. Host instars, N1, N2, N3 and N4 are first, second, third and early fourth instars, respectively. Bar heads with different lowercase letters in each cluster indicate significant differences (HSD test; *P*<0.05) in number of hosts parasitized or fed among different host instars. Bar heads with different capital letters for each instars between the clusters indicate significant differences (HSD test; P<0.05) in number of hosts killed among different host densities. Vertical bars indicate ± one SE.

In mixed-instar host test, at 20 nymphs per cage, a similar number of the four host instars were killed (Figure 3A, *F*_3, 36_  = 2.16, *P* = 0.109). With increased host density, the number of the third and fourth instar nymphs killed by females increased (*F*_3, 36_  = 29.22 and 28.82, respectively, both *P*<0.0001), while that of the first instar decreased (Figure 3A, *F*_3, 36_  = 12.48, *P*<0.0001). There was little change in the numbers of second instar nymphs attacked (Figure 3A, *F*_3, 36_  = 1.94, *P* = 0.140). At 60 and 80 nymphs per cage, female killed most on third and fourth instars, followed by second instar, and least on first instar (Figure 3A, *F*_3, 36_  = 49.61 and 66.89 at 60 and 80 nymphs per cage, respectively, both *P*<0.0001).

Numbers of different instar nymphs parasitized by *En. sophia* females varied significantly at the four host densities (Figure 3B, *F*_3, 36_  = 19.75, 7.04, 29.03 and 43.59, at 20, 40, 60 and 80 nymphs per cage, respectively, *P*<0.0008 for each). The females parasitized most on third and fourth instars, followed by second, and least on first intars at 20, 60 and 80 nymphs per cage, while parasitized similar number of third, fourth and second insta r, and less number of first instar at 40 nymphs per cage (Figure 3B). The numbers of parasitized third and fourth instar nymphs increased significantly (*F*_3, 36_  = 11.59 and 14.86, respectively, both *P*<0.0001) with increased host density, while the numbers of parasitized first (*F*_3, 36_  = 2.20, *P* = 0.104) and second (*F*_3, 36_  = 1.95, *P* = 0.140) instar nymphs did not differ (Figure 3B). As the host density increased from 20 to 40, the number of each instar parasitized were not significantly increased (Figure 3B), however, the total increase in parasitism (1.4 nymphs) was mainly due to the increased number of second instars parasitized (0.7 nymphs).

At the host density of 20 nymphs per cage, *En. sophia* females fed most on the first instar nymphs, followed by second, third and fourth instars with similar numbers (Figure 3C, *F*_3, 36_  = 5.88, *P* = 0.002). At 40 nymphs per cage, the number of third instar nymphs fed by females increased significantly from the lower host density (Figure 3C, *F*_3, 36_  = 16.52, *P*<0.0001). The total increase in host feeding (1.4 nymphs) was mainly due to the increased number of third (1.0 nymphs) and fourth (0.3 nymphs) instars. With the host density increased to 60 and 80 nymphs per cage, females increasingly allocated their host feeding from first instar to third and fourth instars (Figure 3C, *F*_3, 36_  = 10.30, 16.52 and 9.69 for N1, N3 and N4, respectively, *P*<0.0001 for each). At 60 nymphs per cage, *En. sophia* female fed most on third instar and fourth instars (Figure 3C, *F*_3, 36_  = 11.21, *P*<0.0001). At 80 nymphs per cage, *En. sophia* female fed most on third instar nymphs (Figure 3C, *F*_3, 36_  = 14.90, *P*<0.0001).

### Host-feeding ratio in single-instar & mixed-instar host tests

**Figure 4:**
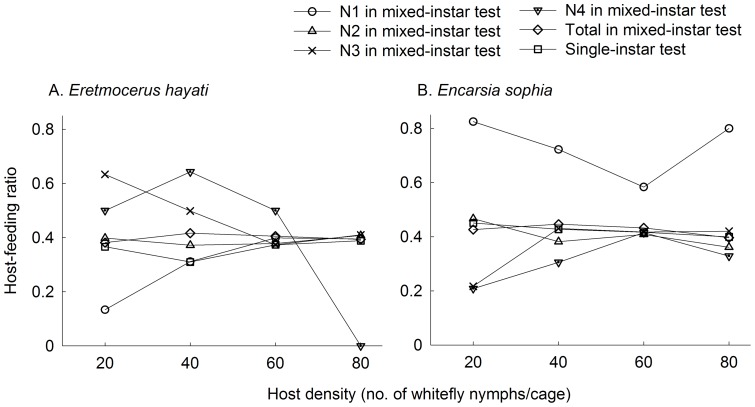
Mean proportion of accepted hosts fed upon by parasitoid in single-instar and mixed-instar host experiment. A, B: Proportion of hosts fed upon in 24h by 1 d-old females of *Eretmocerus hayati* or *Encarsia sophia*, respectively. An area of 3.5 cm^2^ of a leaf on a potted tomato plant was covered by a clip cage. Host instars, N1, N2, N3 and N4 are first, second, third and early fourth instars, respectively. Single-instar tests were performed on N2 for *Er. hayati*, and N3 for *En. sophia.*

In single-instar no choice tests, the host-feeding ratio (proportion of hosts fed upon to total hosts accepted either to parasitize or feed) of *Er. hayati* initially declined with increased host density, then increased and leveled off (Figure 4A, *χ^2^* = 26.43, *df*  = 8, *P* = 0.001), while that of *En. sophia* did not differ (Figure 4B, *χ^2^* = 6.91, *df*  = 8, *P* = 0.547). At 80 nymphs per cage, the host-feeding ratio of *Er. hayati* and *En. sophia* was 0.39 (SE  = 0.01) and 0.40 (SE  = 0.02), respectively. At lower host densities (5–60 nymphs per cage), the hosts feeding ratio of *En. sophia* was significantly higher than that of *Er. hayati* (*Mann-Whitney U* = 24.0, 15.0, 9.5, 6.0, 4.0 and 1.0, *P* = 0.047, 0.007, 0.002, 0.001, 0.0005 and 0.0002, at 5, 10, 20, 30, 40, 50 nymphs per cage, respectively).

In mixed-instar host choice test, the host-feeding ratio of *Er. hayati* on first instars (*χ^2^* = 12.22, *df*  = 3, *P* = 0.007) initially increased with increased host density, then declined and leveled off, while on third (*χ^2^* = 4.42, *df*  = 3, *P* = 0.220) and second instars (Figure 4A, *χ^2^* = 0.62, *df*  = 3, *P* = 0.891) did not differ. The host-feeding ratio in total in mixed-instar choice condition was significantly higher than that in single-instar no-choice condition at 40 nymphs per cage (*Mann-Whitney U* = 9.00, *P* = 0.002), while the differences were not significant at other experimental densities (Figure 4A, *Mann-Whitney U* = 40.0, 34.5 and 47.5, *P* = 0.442, 0. 238 and 0.849, at 20, 60 and 80 nymphs per cage, respectively). At 20 nymphs per cage, the host-feeding ratios varied significantly on different instars (Figure 4A, *χ^2^* = 12.15, *df*  = 3, *P* = 0.007). When host density were higher, the host-feeding ratios on each instar were similar (*χ^2^* = 6.28, *df*  = 3, *P* = 0.099 at 40 nymphs per cage; *χ^2^* = 0.685, *df*  = 3, *P* = 0.877 at 60 nymphs per cage) except on the fourth instar at 80 nymphs per cage (Figure 4A, *χ^2^* = 12.32, *df*  = 3, *P* = 0.006).

The host-feeding ratios of *En. sophia* with respect to all of the four instars in mixed-instar choice condition were similar as the third instar in single-instar test (Figure 4B, *Mann-Whitney U* = 39.5, 38.0, 45.0 and 46.5, *P* = 0.651, 0. 363, 0.704 and 0.790, at 20, 40, 60 and 80 nymphs per cage, respectively). At 20 nymphs per cage, the host-feeding ratio on first instar was the highest, followed by the second, and lowest on the third and fourth instars (Figure 4B, *χ^2^* = 20.70, *df*  = 3, *P* = 0.0001). At 40 nymphs per cage, the host-feeding ratio remained highest on first instar, but became lowest on the fourth (Figure 4B, *χ^2^* = 15.42, *df*  = 3, *P* = 0.001). When host density further increased, the host-feeding ratios were similar on each instar (Figure 4B, *χ^2^* = 2.24, *df*  = 3, *P* = 0.534 at 60 nymphs per cage; *χ^2^* = 6.39, *df*  = 3, *P* = 0.094 at 80 nymphs per cage).

## Discussion

Both *Er. hayati* and *En. sophia* females exhibited a tendency to increase both oviposition and host-feeding with increased host density within a certain range. This was observed in other species of parasitoids [24]–[26]. However, the host handling strategy related to varied host densities of *Er. hayati* and *En. sophia* showed differences. In single-instar tests, the number of hosts parasitized by *Er. hayati* increased faster than the number of hosts fed, and reached the maximal number at lower host densities than host-feeding. For *En. sophia*, the two behaviours changed similarly to increased host densities, but the number of hosts fed reached a plateau at lower host densities than the number of hosts parasitized. In the present mixed-instar tests, when host density initially increased, most of the increase in parasitism by *En. sophia* occurred on second instar nymphs, while more than 70% of the increase in host-feeding occurred on third instar which was the optimal instar either for parasitizing or host feeding. In the case of *Er. hayati*, under increased host density, the majority of increase in both parasitism and host-feeding occurred on the first instar, even if the number of first instars fed upon was not the highest. The differences between these two parasitoids may be explained by the egg load of the females. To get nutrients to make more mature eggs is one of the reasons for host feeding [2]–[4]. The number of mature ova of 1-d-old *Er. hayati* was higher than that of *En. sophia* (Yang NW, unpublished data). Consequently, more eggs could be laid by *Er. hayati* with less host-feeding than *En. sophia*, and the priority for host-feeding in *En. sophia* seems reasonable.

In the present study, the proportion of each host instars killed by these two parasitoids varied with different host densities. At low host density, the host encounter rate is also low [4], which possibly forces the parasitoid to compromise and use not only the favorite, but also other host instars. However, the host-feeding ratio on alternative instars was higher than that on optimal instars, suggesting that parasitoid females partition their feeding and oviposition behaviour on different instars as expected on theoretical grounds, and the optimal instars for oviposition were fed upon less [3], [10]–[13]. At high host density, the host encounter rate is also high [4], providing opportunity to exercise instar preferences without compromise. Under those conditions, more optimal instars than alternative ones were killed, but the host-feeding ratio on each instar was similar. This indicates that there was no partition between feeding and oviposition towards different instars. Host-feeding ratio of *Er. hayati* in the single-instar condition was a little lower than that under mixed-instar presence, and the availability of mixed instars had a smaller effect on host-feeding ratio than that of host density.

Since the host handling strategy varied with host density, as well as by species, the assessment of the preferred instar for oviposition vs. host-feeding needs to be made with caution. When host resources included a mixture of different instars and were abundant, both parasitoids oviposited, but also host-fed mostly on the optimal instars, which obey to their true preference. In the case of limited host resources, host-feeding was switched onto alternative instars, but in a species-specific manner. Thus, under a mixed-instar host availability scenario, the oviposition response is conservative, but the preferred instars for host-feeding could not simply be identified by the number of each instar fed upon since the shortage of suitable instars at low density drives the females to shift the true preference. One has to make sure that the host density is high enough not to compel the females to switch host-feeding on alternative instars – under limited host availability, these will be the suboptimal ones, masking their true preference. This is not yet obvious in the literature, because most studies were done under a single (even though usually high) host density [8], [23], [27]. Host-feeding behaviour observed under single-instar host availability may also differ from the more natural, mixed-instar host presence. *En. sophia*, *En. formosa* and *Eretmocerus melanoscutus* (Zolnerowich & Rose) (Hymenoptera: Aphelinidae) host-feed most on first and second instar nymphs of *B.tabaci* Middle East-Asia Minor 1 in single-instar experiments, while third and fourth instar nymphs are fed upon most when a mixture of different instars are available [23]. Since the co-occurrence of different-instar nymphs on one leaf is the common condition in the field, host-feeding results obtained using a single instar may not well predict the real biocontrol efficiency by host-feeding in the field.

Our findings have consequences for the practice of biological control, especially for augmentative releases. It is very important to find ways to manipulate the parasitoids so that they quickly destroy as many hosts as possible through destructive host feeding and parasitism. The present study suggests that the combined release of these two parasitoids might have better control on *B. tabaci* than that of either species alone, since these two parasitoids not only preferred different host instars to oviposit and host-feed at high host density but also reacted differently to increased host densities. Releasing one species might be efficient at low host densities since oviposition and host-feeding under those conditions would be partitioned on different host instars and no instar will be free of parasitoid pressure. However, when host density is high, attack will shift onto the favorite host instar/s. In such cases, the release of multiple parasitoids which complement each other via differences in preferred instars might be more effective than repeated single parasitoid releases. However, as *En. sophia* is an autoparasitoid, the interference to *Er. hayati* population by the male producing hyperparasitizing behavior when released together need to be concerned.

Optimal foraging models predict that parasitoid females oviposit on hosts of high quality, while feed on the ones of lower quality [3], [10]. Our study demonstrates that the preference and intensity of oviposition and host-feeding varied with host density. Organisms are under environmental constraints that limit their possibilities to maximize their fitness [28]. Our findings suggest that once the food resource stress is relaxed, the parasitoid strategy of allocating host recourses increases the fitness not only via reproduction, but also via body maintenance. However, the difference in the reaction of females released in patches with different host densities may be not only numerical, further behavioral observation need to be conducted to reveal the mechanism behind this.
